# Peripheral Nerve Regeneration: Mechanism, Cell Biology, and Therapies

**DOI:** 10.1155/2014/145304

**Published:** 2014-12-23

**Authors:** Xiaofeng Jia, Mario I. Romero-Ortega, Yang D. Teng

**Affiliations:** ^1^Department of Neurosurgery, University of Maryland School of Medicine, 10 South Pine Street, Medical School Teaching Facility 559 A, Baltimore, MD 21201, USA; ^2^Department of Biomedical Engineering, Physical Medicine & Rehabilitation, Anesthesiology & Critical Care Medicine, Johns Hopkins University, Baltimore, MD 21205, USA; ^3^Department of Bioengineering, The University of Texas at Dallas, Richardson, TX, USA; ^4^Surgery Department, University of Texas Southwestern Medical Center, TX, USA; ^5^Departments of Neurosurgery and Physical Medicine & Rehabilitation, Harvard Medical School, Brigham and Women's Hospital, Spaulding Rehabilitation Hospital and VA Boston Healthcare System, Boston, MA, USA

Peripheral nerve injury has perplexed neuroscientists, neurologists, plastic surgeons, and bioengineers for decades. Despite the spontaneous ability of the adult peripheral nerve system to regenerate after injury, optimal and universal treatments capable of achieving full functional recovery are still unavailable. It is the suboptimal results obtained with current gold standard autograft surgery methods that continue to motivate vigorous research around the world for establishing biosynthetic and/or cell-based alternatives, aiming at elucidating the underlying mechanisms and developing new regenerative medicine approaches in order to maximize functional nerve regeneration and to prevent occurrence of neuropathic pain.

In this special issue, six papers are published that provide a global view of the challenges in peripheral nerve repair research and therapeutic development: from advanced biomaterial research to the resolution overcoming clinical practice limitations, which bears the promise of improving clinical outcomes for patients with nerve injuries.

D. Grinsell and C. P. Keating presented a current view of the clinical results achieved with peripheral nerve regeneration using nerve conduits and synthetic bonding materials. E. P. Knott and collaborators elaborated on recent advancement regarding the molecular response of peripheral nerves to injury insults, investigating a specific connection of the intracellular cyclic adenosine monophosphate (cAMP) cascade and its modulation that might help overcome molecular inhibitors of axonal growth to serve as a strategy for PN regeneration. For the biomaterials that have been shown or proposed to activate growth-promoting responses after nerve injury, P. Ramburrun et al., in their paper, demonstrated a comprehensive strategy that was used to achieve sustained release of bioactive molecules from nerve conduits as a possible clinical application for peripheral nerve injury repair. The authors gave careful consideration to critical aspects of the delivery of biologically active factors into the injured nerve and presented state-of-the-art chemical engineering methods to ensure long-term controlled release of therapeutic reagents. Electrical stimulation is one of the methodologies that have been conventionally used to overcome the progressive loss of the nerve regenerative capacity when lesions occur far from the target organ. D. C. Miranda de Assis et al. compared the role of stimulation frequency on the use of transcutaneous nerve stimulation (TENS) as a method to stimulate peripheral nerve regeneration and reported confirmative experimental data.

Besides functional deficits, almost all types of nerve lesions could result in development of neuropathic pain. In this issue, V. Magnaghi and collaborators reported that an agonist-mediated modulation of the gamma-aminobutyric acid (GABA) system showed efficacy for ameliorating neuropathic pain. Finally, the report by H. Su et al. showed that administration of lithium, normally used for the treatment of mood disorders such as bipolar disorders and depression, was beneficial for the nerve regrowth in a model of brachial plexus root avulsion. The study demonstrated that the lithium treatment reduced metabolic demand of motor neurons and increased motor neuron survival after root avulsion injury.

It is our hope that the compilation of these research reports would provide an updated view of regenerative biology of peripheral nerve injury as well as academic insight that may facilitate further improvement of current clinical protocols for peripheral nerve repair. Most importantly, we wish that new data published here would enable development of novel ideas to move our field forward.

